# Device Design Modifications Informed by In Vitro Testing of Bacterial Attachment Reduce Infection Rates of Cochlear Implants in Clinical Practice

**DOI:** 10.3390/microorganisms9091809

**Published:** 2021-08-25

**Authors:** Lynne Turnbull, Roger Leigh, Rosalia Cavaliere, Sarah R. Osvath, Laura M. Nolan, Daniel Smyth, Kristien Verhoeven, Richard A. Chole, Cynthia B. Whitchurch

**Affiliations:** 1 The iThree Institute, University of Technology Sydney, Ultimo, NSW 2007, Australia; Lynne.Turnbull@leica-devices.com (L.T.); rosalia.cavaliere@uts.edu.au (R.C.); Sarah.Osvath@uts.edu.au (S.R.O.); 2 Cochlear Limited, 1 University Avenue, Macquarie University, Sydney, NSW 2109, Australia; rleigh@cochlear.com (R.L.); dsmyth@cochlear.com (D.S.); kverhoeven@cochlear.com (K.V.); 3 National Heart and Lung Institute, Imperial College London, London SW3 6LR, UK; l.nolan@imperial.ac.uk; 4 Washington School of Medicine in St Louis, Washington University in St. Louis, St. Louis, MO 63110, USA; rchole@wustl.edu; 5 Quadram Institute Bioscience, Norwich Research Park, Norwich NR4 7UQ, UK; 6 School of Biological Sciences, University of East Anglia, Norwich NR4 7TK, UK

**Keywords:** biofilm, medical device, infection, antibiotic resistance, AMR

## Abstract

Recalcitrant chronic infections of implanted medical devices are often linked to the presence of biofilms. The prevention and treatment of medical device-associated infections is a major source of antibiotic use and driver of antimicrobial resistance globally. Lowering the incidence of infection in patients that receive implanted medical devices could therefore significantly improve antibiotic stewardship and reduce patient morbidity. Here we determined if modifying the design of an implantable medical device to reduce bacterial attachment, impacted the incidence of device-associated infections in clinical practice. Since the 1980s cochlear implants have provided long-term treatment of sensorineural hearing deficiency in hundreds of thousands of patients world-wide. Nonetheless, a relatively small number of devices are surgically explanted each year due to unresolvable infections. Features associated with the accumulation of bacteria on the Cochlear™ Nucleus^®^ CI24RE™ model of cochlear implant devices were identified using both in vitro bacterial attachment assays and examination of explanted devices. Macro-scale design modifications that reduced bacterial attachment in vitro were incorporated into the design of the CI500™ and Profile™ series of Nucleus implant. Analyses of mandatory post-market vigilance data of 198,757 CI24RE and 123,084 CI500/Profile series implantation surgeries revealed that these design modifications correlated with significantly reduced infection rates. This study demonstrates that a design-centric approach aimed at mitigating bacterial attachment was a simple, and effective means of reducing infections associated with Cochlear Nucleus devices. This approach is likely to be applicable to improving the designs of other implantable medical devices to reduce device-associated infections.

## 1. Introduction

Over half of healthcare-associated infections can be attributed to implanted medical devices occurring in 0.08–50% for the most common implant types depending on the implant site, how long the device is implanted, patient comorbidities, or other risk factors with the highest rates of infection associated with urinary and cardiac devices [1,2]. Device-related infections are difficult and costly to treat due to lengthy antibiotic therapy and may require surgical intervention and device removal in cases where the infection cannot be resolved with antibiotics [1]. Recalcitrant chronic infections of implanted medical devices are often linked to the presence of persister cells [3] and surface-attached communities of microorganisms called biofilms that have elevated resistances to antibiotics and the immune system [4]. Almost immediately after a medical device is implanted it becomes coated with a conditioning film comprised of host extracellular matrix, plasma proteins, and platelets which can serve as receptors for colonizing bacteria and fungi which adhere to the implant surface and establish biofilms [5].

The cochlear implant has been used to treat many forms of sensorineural hearing deficiency in children as young as 5 months old to seniors in their 90s [6]. The component that houses the receiver and stimulator circuitry is surgically implanted beneath the skin behind the ear and secured in place [7]. The Cochlear™ Nucleus^®^ device has two leads, one of which contains an array of 22 electrodes that is inserted into the cochlea to stimulate auditory nerves and a second lead which serves as a ground [7]. Following surgical implantation the cochlear implant becomes encapsulated in fibrotic scar tissue [8].

Occasionally, days to even years later, chronic bacterial infections at the site of the cochlear implant occur that are resistant to antimicrobial therapy [9]. These infections can lead to tissue destruction, device dysfunction, and systemic dissemination of the pathogen. In extreme cases, chronic and recurrent infections that are recalcitrant to therapeutic intervention require device removal and subsequent re-implantation surgeries with associated trauma [10]. While cochlear implant devices have proven to be highly successful, a relatively small number are surgically removed each year due to unresolvable infections. It is imperative that every effort should be made to reduce the incidence of device-associated infections to minimize the risks and burdens to cochlear implant recipients.

The aim of this study is to assess if altering the macro-scale design of the Cochlear Nucleus device, to ameliorate the potential for bacterial attachment, could reduce the incidence of device-associated infections requiring surgical removal of the implant. Our aim was to identify sites on the Cochlear Nucleus device which were prone to bacterial attachment and use this data to inform modifications of the device design to mitigate bacterial attachment. We utilized a combination of in vitro bacterial attachment assays and direct examination of explanted devices to identify features of the Nucleus CI24RE™ model of cochlear implant that were associated with bacterial attachment. We then modified the design of these features in an attempt to mitigate bacterial attachment. The efficacy of these design modifications was tested on prototype devices using our in vitro bacterial attachment assay. The design modifications that were associated with reduction in bacterial attachment in vitro were subsequently incorporated into the design of the Cochlear Nucleus implant model CI500™ series (in use from 2010–2011) and the Profile™ series (in use from 2014-present). A retrospective analysis of mandatory post-market vigilance data was performed to determine the impact of these design modifications on infection rates.

## 2. Materials and Methods

### 2.1. Identification of Bacterial Biomass on Cochlear Implant Devices In Vitro

Cochlear implant devices used in this study were provided by the manufacturer Cochlear Limited, Sydney, Australia. *Staphylococcus aureus* is frequently isolated from wound infections at the site of cochlear device implantation [10]. The *S. aureus* strain (CI494) used in this study was isolated from the site of a cochlear implant-associated infection and obtained from St Vincent’s Hospital, Melbourne, Australia. To identify features that promoted bacterial attachment, in vitro bacterial attachment assays were performed on ten CI24RE model Nucleus cochlear implant devices. *S. aureus* was allowed to attach to cochlear implant devices suspended in nutrient broth for 48 h, washed to remove loosely adherent bacteria, stained and visually inspected by fluorescence microscopy to determine sites of bacterial attachment as follows. Sterile Cochlear Nucleus devices were coated for 24 h in fetal calf serum at 4 °C to provide a conditioning film for *S. aureus* attachment. The devices were then suspended in 1:10,000 dilution of an overnight culture of *S. aureus* in tryptic soya broth (TSB) and incubated with gentle stirring at 37 °C for 48 h with a change of media at 24 h. The devices were then washed three times in sterile phosphate buffered saline (PBS) to remove loosely adherent bacteria. Attached *S. aureus* cells were stained with the cell permeant DNA stain Syto 9 (2.5 μM; Thermo Fisher Scientific Australia, Scoresby, VIC, Australia) and visualized with epifluorescence on an Olympus IX71 inverted research microscope with a FViewII monochromatic camera (Olympus Australia, Notting Hill, VIC, Australia) and AnalySIS Research acquisition software (version 2; Olympus Australia, Notting Hill, VIC, Australia).

### 2.2. Cochlear Implant Device Design Modifications

Having identified features of the CI24RE device that were common sites of bacterial attachment, design modifications were incorporated into the CI500/Profile series in an attempt to mitigate bacterial attachment in these regions. These modifications included ensuring clean cut silicone edges, removal of raised lettering, and the use of wider recesses with smoother transitions. The same biocompatible materials used in the CI24RE devices were used in the CI500/Profile devices. To determine if these design modifications reduced bacterial attachment in vitro, bacterial attachment assays were performed as described above with four CI500/Profile model prototypes and four CI24RE devices concurrently as matched comparison controls.

### 2.3. Identification of Bacterial Biomass on Explanted Cochlear Implant Devices

The post-market vigilance process requires all explanted cochlear implant devices to be returned to the manufacturer for mechanical and electrical assessment. This process standardly involves sterilization and removal of all biological material and is therefore not compatible with determining sites of bacterial biomass. We were interested in determining if and where bacterial biomass was located on devices that had been explanted due to infection or electro-mechanical failure. To achieve this, a small number of devices were processed immediately after explant as follows. Four CI24RE devices from patients with unresolvable infection at the cochlear implant site and two CI24RE devices from patients with electro-mechanical failures of the cochlear implant device and no clinical evidence of infection were surgically removed at the Washington University School of Medicine in St Louis, USA and placed immediately into sterile jars containing a formalin/ethanol solution (50% (*v*/*v*) of a 10% solution of neutral buffered formalin (NBF): 45% (*v*/*v*) absolute ethanol and 5% (*v*/*v*) glycerol). The explanted devices were then couriered to our laboratory in Sydney, Australia for analysis of bacterial biomass by fluorescence in situ hybridization (FISH). The oligonucleotide FISH probe used in this study (synthesized by Integrated DNA Technologies, Inc. Singapore 117610, Republic of Singapore) was the general eubacterial probe EUB-388-Cy3 (5′-/5Cy3/GCT GCC TCC CGT AGG AGT3′) [11].

Detection of Gram-negative bacterial biomass by FISH was performed as follows. Formalin/ethanol fixed devices were passed through an ethanol dehydration procedure (50% (*v*/*v*), 80% (*v*/*v*), 95% (*v*/*v*) ethanol, 30 min each) and allowed to dry at room temperature. For general detection of Gram-negative bacteria, samples were hybridized with the 16S rRNA oligonucleotide probe (EUB-388-Cy3) as follows. Hybridization buffer (0.9 M NaCl, 0.01% (*v*/*v*) SDS, 20 mM Tris-HCl pH 7.2, formamide 20% (*v*/*v*)) containing 10 ng/μL of FISH probe was applied to the samples before placing them in a humidified chamber at 46 °C for 3 h. Samples were then de-stained with a wash buffer (20 mM Tris-HCl pH 7.2, 0.01% (*v*/*v*) SDS, 40 mM NaCl, 5 mM ETDA) for 15 min at 46 °C in a humidified chamber. After washing with MilliQ water, samples were air dried at room temperature. Samples were imaged using a DeltaVision Elite inverted research microscope with InsightSSI illumination, SoftWorX acquisition software, fitted with a scientific CMOS 15-bit camera (pco.edge, PCO AG, Kelheim, Germany).

The Gram-negative FISH staining procedure does not enable penetration of the FISH-probe into Gram-positive bacteria due to their thick cell-walls. Therefore, after imaging for Gram-negative bacteria, devices were then treated with 10 μg/mL of Proteinase K and incubated at 37 °C for 1 h. The reaction was then stopped with ice-cold PBS. This Proteinase K step removes Gram-negative bacteria due to destruction of the thin Gram-negative cell wall and allows the FISH probe to enter Gram-positive cells so that only Gram-positive bacterial biomass is visualized. Hybridization of the 16S rRNA oligonucleotide probe (EUB-388-Cy3) and fluorescence microscopy were performed as described for detection of Gram-negative bacteria. Following completion of the microscopic assessment of bacterial burden, the devices were returned to the manufacturer to enter the routine returned devices assessment process.

### 2.4. Analysis of Outcomes in Clinical Practice

Mandatory post-market surveillance and vigilance data were collected according to regional-specific regulations. This ensures a high level of compliance and reporting of outcomes associated with all Cochlear Nucleus implant surgeries. Post-market surveillance and vigilance data were obtained for 198,757 CI24RE implantation surgeries from 2005 until 2020, 29,895 CI500 implantation surgeries from 2010–2011 and 93,189 Profile implantation surgeries from 2014 until 2020. Complaints and issues reported for implanted Cochlear Nucleus devices before (CI24RE series) and after (CI500/Profile series) design modifications were compared, and statistical analyses performed to assess differences in the reported rates of unresolvable infections leading to device removal. Specifically, post-market vigilance data were examined for reports of infections that were recalcitrant to antibiotic therapy and required surgical removal of the device. These were classified as “infections leading to explant”. As both the CI500 and Profile series devices have identical external designs and materials, in this study we refer to both of these devices collectively as “CI500/Profile” unless the data refers to only the CI500 or the Profile separately.

### 2.5. Statistical Analyses

Statistical analyses were performed using Minitab software [12]. Actuarial analyses were performed with 12-month analysis intervals since implantation and the survival probability is presented with 95% two-sided confidence intervals. Survival curves were compared with Log-Rank and Wilcoxon tests.

## 3. Results

**Figure 1:**
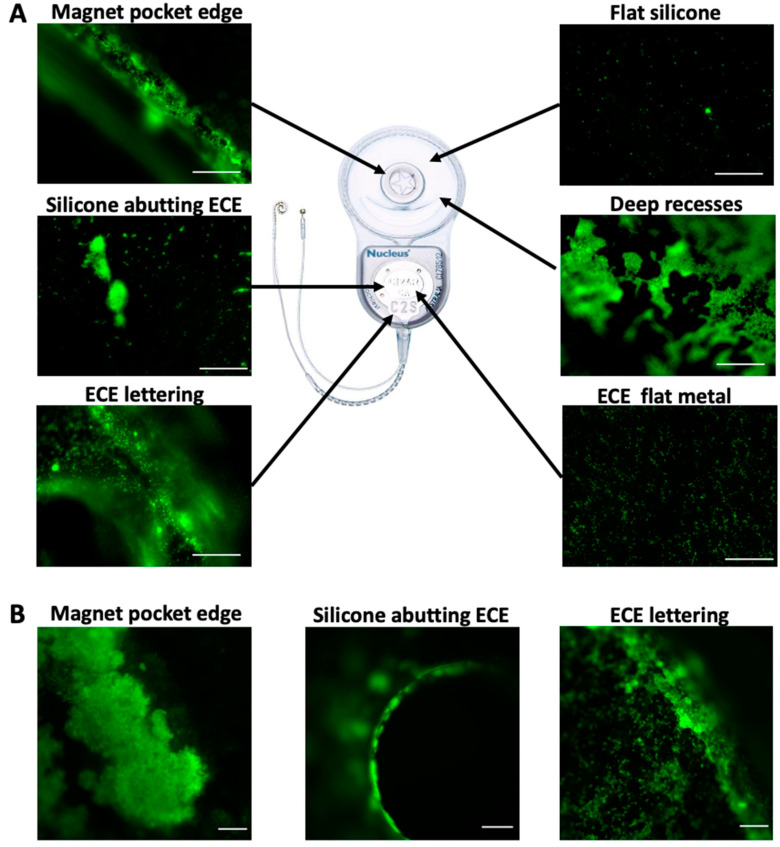
Imaging of bacterial biomass on cochlear implant devices. (**A**) Attachment of *S. aureus* to CI24RE devices in in vitro assays visualized with Syto-9 (green) stain. (**B**) CI24RE devices explanted due to infection visualized using a general eubacterial FISH probe (green). Imaged regions are as follows: magnet pocket edge, silicone abutting extracochlear electrode (ECE), ECE lettering, ECE flat metal, deep recesses, or flat silicone. Scale bar is 100 µm for all images. Images are representative of (**A**) ten or (**B**) four devices.

We first performed in vitro bacterial attachment assays to identify features of the Cochlear Nucleus CI24RE model that routinely acquired higher bacterial biomass compared to other regions of the device. Features that were associated with higher levels of bacterial attachment in vitro included rough silicone edges such as those at the edge of the magnet pocket and abutting the extracochlear electrode (ECE) plate; the metal surface and raised lettering of the ECE plate; and regions with deep recesses and steep sides (Figure 1A). Flat areas of silicone had fewest attached bacteria (Figure 1A).

We also examined if and where bacterial biomass was located on CI24RE devices explanted due to unresolvable infections. We found that bacterial biomass was located on the ECE plate (silicone edges and raised lettering) in all four CI24RE devices associated with infections and on the silicone edges of the magnet pocket in three of the four devices (Figure 1B). Therefore, there appeared to be bacterial biomass and biofilms situated in the same regions of CI24RE devices that had been surgically removed due to infection as had been identified in our in vitro assay as areas to which bacteria had a propensity to attach. There was little to no bacteria present on the devices that had been surgically removed due to electro-mechanical failure, indicating that the presence of bacterial biomass on the devices associated with infections were unlikely to be an artefact of the staining procedure.

**Figure 2:**
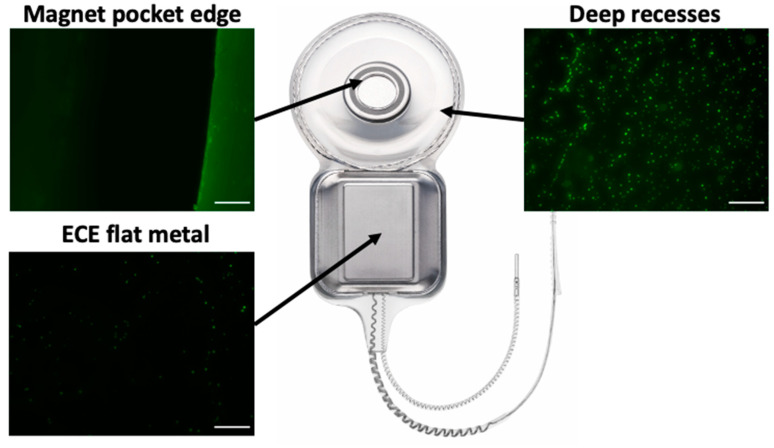
Bacterial attachment is decreased on CI500/Profile series cochlear devices. Attachment of *S. aureus* to prototype CI500/Profile series devices in in vitro assays visualized with Syto-9 (green) stain. Imaged regions were the magnet pocket edge, deep recesses or extracochlear electrode (ECE) flat metal. All regions showed very little bacterial attachment and no bacterial aggregates were observed. Images of CI24RE devices imaged at the same time as these CI500/Profile images were identical to those shown in Figure 1A. Scale bar is 100 µm. Images are representative of four devices.

Having identified features of the CI24RE cochlear implant that appeared to promote bacterial attachment which could potentially lead to unresolvable infections, we modified some of these features of the CI500/Profile series in an attempt to mitigate this. We performed in vitro bacterial attachment assays of CI500/Profile series prototypes incorporating these design modifications. Visual inspection of these prototype devices showed reduced levels of bacterial attachment in these regions compared to CI24RE devices assayed concurrently (Figure 2).

**Figure 3:**
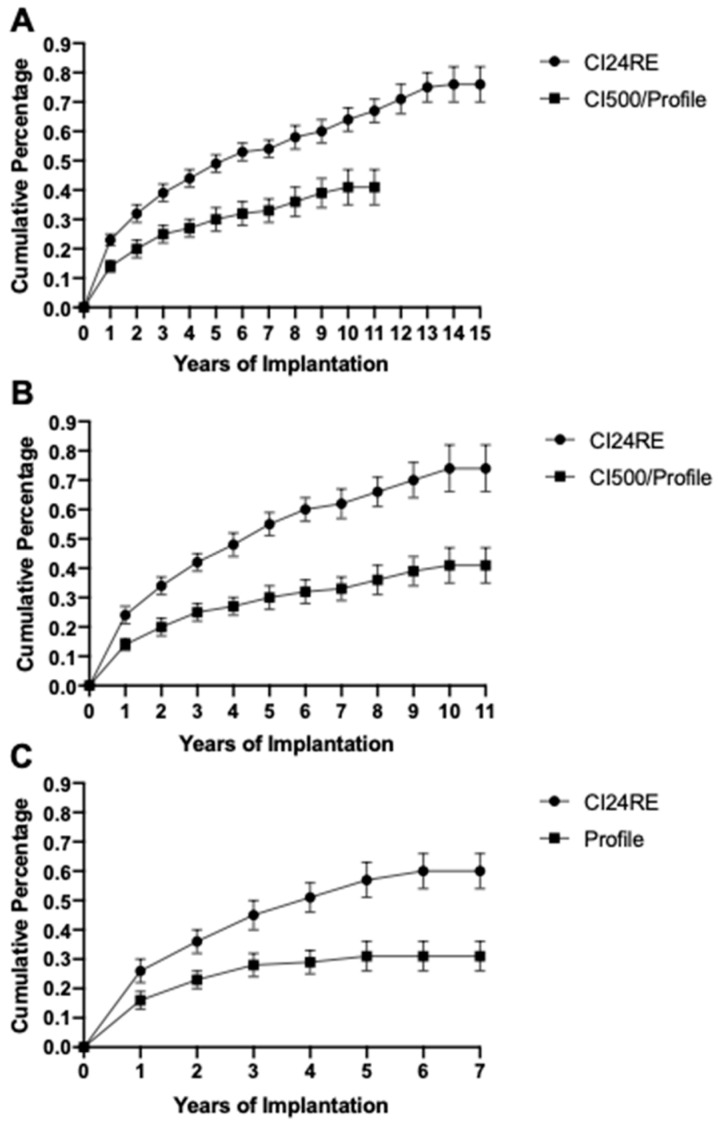
Cumulative percentage of infections leading to explant per year of implantation for Cochlear Nucleus devices before (CI24RE) and after (CI500/Profile) design modifications. Cumulative percentages of infections leading to explant per year since device implantation for (**A**) CI24RE (devices implanted from 2005–2020) or CI500/Profile (devices implanted from 2010–2020); (**B**) CI24RE or CI500/Profile (devices implanted from 2010–2020); (**C**) CI24RE or Profile (devices implanted from 2014–2020). Data are presented as the cumulative failure percentage with 95% confidence intervals. For (**A**) CI24RE (*n* = 198,757) or CI500/Profile (*n* = 123,084), (**B**) CI24RE (*n* = 142,439) or CI500/Profile (*n* = 123,084) and (**C**) CI24RE (*n* = 80,845) or Profile (*n* = 93,189).

The design modifications that reduced bacterial attachment in vitro were incorporated into the CI500 series of the Nucleus cochlear implant available from 2010–2011 and the Profile model of Nucleus cochlear implant available from 2014-present. Post-market vigilance data of 198,757 CI24RE and 123,084 CI500/Profile series implantation surgeries from 2005–2020 was examined for reports of infections that were recalcitrant to therapy and required surgical removal of the device. Analysis of this infection data revealed that patients who received the CI500/Profile model had significantly lower overall rates of infection leading to device explant compared to those that had received the CI24RE device (Figure 3A; *p*-value of <0.0005 for Log-Rank and Wilcoxon tests demonstrating statistically significant differences). To assess if the differences in these infection rates are due to alterations in the implanted Nuclear device design rather than other influencing factors such as changes in clinical sterility, surgeon training and guidelines, and antibiotic availability and usage practices, we compared infection data of patients who received either the CI24RE or CI500/Profile series across the same time periods. Data were obtained for patients who received either the CI24RE (142,439) or CI500/Profile series (123,084) from 2010–2020 (Figure 3B) and for patients who received either the CI24RE (80,845) or Profile (93,189) cochlear implant device from 2014–2020 (Figure 3C). Analysis of this data showed that the rates of infection leading to device explant during the same time periods was significantly lower for the CI500/Profile series than for the CI24RE Nucleus model (Figure 3B,C; *p*-values of <0.0005 for Log-Rank and Wilcoxon tests demonstrating statistically significant differences).

To determine if the differences in infection rates for the CI24RE and CI500/Profile devices were due to factors other than device design, analysis of available demographic information on the implant recipients was performed. The clinical indications for use are the same for both the CI24RE and CI500/Profile devices and both devices have been implanted in over 100 countries. The infection rate data for the implant periods 2010–2020 (Figure 3B) and 2014–202 (Figure 3C) were reviewed for countries in each of the Americas, Europe, and Asia-Pacific regions as well as the G7 countries. In all geographical regions, the rates of infection leading to explant were significantly reduced for the CI500/Profile series compared to the CI24RE (Table 1 and Table 2).

The data were also analyzed according to recipient gender and age. For the devices implanted in 2010–2020 (Figure 3B), the proportion of pediatric recipients of the CI24RE device is 56.2% while the proportion of pediatric infections with the CI24RE is 57.4%. The proportion of pediatric recipients of the CI500/Profile is 40.0% while the proportion of pediatric infections with the CI500/Profile is 36.3%. For this period, the proportion of female recipients of the CI24RE is 47.3% while the proportion of female infections with the CI24RE is 47.7% and the proportion of female recipients of the CI500/Profile is 48.3% while the proportion of female infections with the CI500/Profile is 46.5%.

For the devices implanted in 2014–2020 (Figure 3C), the proportion of pediatric recipients of the CI24RE is 61.3% while the proportion of pediatric infections with the CI24RE is 60.5%. The proportion of pediatric recipients of the CI500/Profile is 39.6% while the proportion of pediatric infections with the CI500/Profile is 31.7%. For this period, the proportion of female recipients of the CI24RE is 46.3% while the proportion of female infections with the CI24RE is 45.5% and the proportion of female recipients of the CI500/Profile is 47.7% while the proportion of female infections with the CI500/Profile is 45.7%.

In general, the infection rate proportion follows the age and gender demographic proportions except for the pediatric infection rate for CI500/Profile which is lower than the proportion of recipients. As the proportion of pediatric recipients for the CI500/Profile is less than 50% this will to some extent counter the lower infection rate overall. Despite this effect, the lower infection rate of CI500/Profile compared to CI24RE is significant.

These analyses indicate that the significant improvement in rates of infections leading to explant of the CI500/Profile compared to the CI24RE series of Nucleus cochlear implants is likely to be due to the differences in device design, and not due to other demographic factors associated with geographical region, age, or gender.

## 4. Discussion

Infections associated with implanted medical devices are often recalcitrant to antimicrobial therapy due to the presence of biofilms [4] and persister cells [3] that have elevated resistances to antibiotics and immune clearance. The overuse of antibiotics to treat device-associated infections is associated with increasing levels of antimicrobial resistance among bacterial pathogens [13]. Therefore, lowering the incidence of infection in patients who receive implanted medical devices will not only result in a reduction of the potential risks and burdens to implant recipients but may also result in reduced antibiotic usage, which is an important consideration for antibiotic stewardship in the fight against antimicrobial resistance. Approaches aimed at preventing bacterial colonization and subsequent infections associated with implanted medical devices largely focus on modifications of the device at the nanoscale. These include alteration of surface properties such as topography, roughness, and hydrophobicity; or involve functionalized surfaces that elute antibiotics; or that incorporate bactericidal moieties or anti-adhesive polymers [14,15]. However, to-date, little consideration has been given to the contribution of device design features at the macro-scale in promoting or inhibiting the propensity of bacteria to initiate attachment to implanted medical devices.

In this study we utilized direct microscopic examination of Cochlear Nucleus implant devices to identify physical features that may promote bacterial attachment and subsequent biofilm formation. We found that modification of the design of the Cochlear Nucleus implant device, while utilizing the same biocompatible materials, was able to mitigate bacterial attachment to these features in vitro. Specifically, we implemented modifications to ensure clean cut silicone edges, removal of raised implant identification lettering, and use of wider recesses with smoother transitions which proved to be effective in mitigating bacterial attachment in vitro. Importantly, these design modifications were incorporated in the CI500/Profile series of Cochlear Nucleus implant and were associated with a significant reduction in the rates of reports of infections leading to explant in patients who received the CI500/Profile devices relative to CI24RE recipients (Figure 3A). Comparison of data of reports of infections leading to explant in patients who received either the CI24RE or the CI500 or Profile devices over the same time periods and in the same regions, suggests that the observed reduction in infection rates are likely to be associated with the design changes implemented, and not to other factors such as patient demographics or changes in clinical procedures in different geographical locations (Figure 3B,C, Table 1 and Table 2).

To our knowledge this is the first research report addressing the consideration of macro-scale device design as a means of reducing the incidence of device-associated infections. Many other silicone and metal-based implants, e.g., defibrillators, heart pumps, catheters, surgical reconstructive components, and prosthetics have intricate design features that may be sites for preferential bacterial attachment [16]. It is therefore worth considering if physical features of other implanted medical devices may also be amenable to design modifications that will reduce the propensity for bacterial attachment and limit subsequent device-associated infections. This would limit the need for extended antibiotic treatments and reduce the costs, risks, and burdens to recipients of implanted medical devices and health-care systems.

## Figures and Tables

**Table 1:** Percentage of infections leading to explant by geographical region (2010–2020).

Region	CI24RE		CI500/Profile		
	Infection Rate ^1^	Proportion ^2^	Infection Rate ^1^	Proportion ^2^	Fold Change
Americas	0.82%	32%	0.33%	40%	2.5
Asia Pacific	0.44%	37%	0.27%	18%	1.6
Europe/Middle East/Africa	0.65%	31%	0.23%	42%	2.8
G7 countries	0.95%	33%	0.32%	64%	3

^1^ Infection leading to explant; ^2^ proportion of total devices of this model implanted in this period.

**Table 2:** Percentage of infections leading to explant by geographical region (2014–2020).

Region	CI24RE		Profile		
	Infection Rate ^1^	Proportion ^2^	Infection Rate ^1^	Proportion ^2^	Fold Change
Americas	0.71%	28%	0.28%	38%	2.5
Asia Pacific	0.33%	47%	0.25%	19%	1.3
Europe/Middle East/Africa	0.55%	25%	0.21%	43%	2.6
G7 countries	0.81%	24%	0.30%	63%	2.7

^1^ Infection leading to explant; ^2^ proportion of total devices of this model implanted in this period.

## Data Availability

The data used in this study are available upon reasonable request from R.L. The data are not publicly available to protect patient privacy.
